# Resources recovery from high-strength human waste anaerobic digestate using simple nitrification and denitrification filters

**DOI:** 10.1016/j.scitotenv.2019.135509

**Published:** 2020-04-10

**Authors:** Brandon Hunter, Marc A. Deshusses

**Affiliations:** Dept. of Civil and Environmental Engineering, 127C Hudson Hall, Box 90287, Duke University, Durham, NC 27708-0287, USA

**Keywords:** Sanitation, Resource recovery, Reuse, Struvite, Nitrification, Denitrification

## Abstract

Simple trickling nitrification filters and submerged denitrification filters were developed to provide post-treatment to high-strength human waste anaerobic digestate with the aims to (i) effectively recover nutrients in a useful form as a fertilizer and to (ii) treat digestate such that it could be reused as flush water in water scarce regions. The tested filter media (biochar, granular activated carbon, rice and coconut husks, bamboo chips, sunflower seeds, and zeolite) are low cost and sustainable materials and can be locally sourced where on-site sanitation facilities are in high demand. Experimental data from laboratory operation with digestate from anaerobic digestion of dog feces and human urine revealed that the filters achieved a combined removal of chemical oxygen demand (COD), total nitrogen (TN), and phosphorus (PO_4_-P) up to 84%, 69%, and 89%, respectively. Post-treatment filters have also demonstrated successful recovery of vital nutrients by precipitating ammonium magnesium phosphate hydrate, a documented valuable slow-release solid fertilizer. These filters have a great potential for advancing access to improved sanitation while simultaneously increasing capacity for small-scale economic agricultural development in geographic regions lacking improved sanitation.

## Highlights

• Low-cost filters were investigated for the treatment of fecal waste digestate.
• 84% of the COD was removed in the GAC medium trickling filter.
• 69% of total N was removed in the biochar medium trickling filter.
• 89% PO_4_-P was removed across the system.
• Ammonium magnesium phosphate hydrate was precipitated in the denitrification filter.

## Graphical abstract

**Figure:**
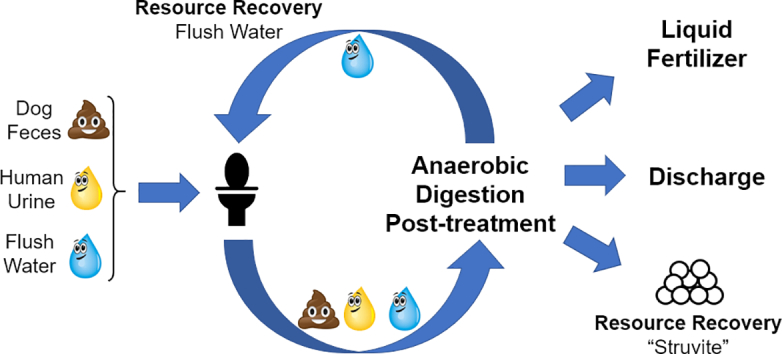

## 1 Introduction

In 2015, the World Health Organization estimated that 4.5 billion people lacked access to safely managed sanitation, of which about 900 million people practiced open defecation (WHO/UNICEF, 2017). It is estimated that about 60% of all fecal waste globally does not undergo any type of treatment (Baum et al., 2013). Because of this, over 50% of all rivers, oceans, and lakes are contaminated with untreated sewage (Mara, 2013). In 2012, over 1.8 billion people were at risk from drinking water sources that were polluted with fecal contaminants (United Nations, 2016). It is estimated that food and water contaminated with fecal matter cause over 2.5 billion diarrheal cases in children five years old or younger each year, resulting in over 500,000 child deaths per year (UNICEF, 2013). The lack of access to safe sanitation has also negative impacts on the economy. The World Bank estimates that inadequate sanitation is responsible for a loss of 6.4% of India's GDP (WSP, 2011). Understanding the dire need to incorporate treatment into sanitation solutions, the United Nations redefined their 2030 goal of achieving universal access to sanitation to also incorporate fecal waste treatment (United Nations, 2015).

However, the implementation of sewer systems in access-deprived areas presents many financial and logistical challenges. Also, the large amounts of water needed for such centralized systems makes them unfit for many places where water is scares. Many areas around the world are increasingly adopting decentralized on-site sanitation (OSS) technologies. Currently, OSS facilities provide sanitation access to over 2.7 billion people globally in low income areas. In urban areas of Ghana and the Philippines, about 85% and 98% of households utilize on-site treatment facilities, respectively (Montangero and Strauss, 2004). Also, it is estimated that 65–100% of all sanitation in Sub-Saharan Africa utilizes on-site sanitation (Strande, 2014). There is a significant need to improve these technologies, as the number of people relying on OSS facilities is expected to increase to 5 billion by 2030 (Strande, 2014).

Anaerobic digestion (AD) is the process of treating organic materials biologically in the absence of oxygen and producing biogas. AD has become a very popular, especially for the treatment of high strength wastes, because it yields an effluent that has significant organic matter reduction (60–80%) as well as producing biogas (about 60% methane and 40% CO_2_) that can be used for cooking or power generation. Anaerobic digestion's cost effectiveness makes it a very attractive treatment for resource-scarce areas. While AD typically does not reduce pathogen to safe levels, a field study conducted by Forbis-Stokes et al. reported that anaerobic digestion of minimally diluted human waste removed about 85–89% of the chemical oxygen demand (COD) (Forbis-Stokes et al., 2016). However, the resulting effluent COD and total ammonia nitrogen (TAN) concentrations of 4500–6500 mg L^−1^ and 2400–4800 mg L^−1^ were sufficiently high that they required further treatment in order to meet acceptable discharge levels should these effluents not be reused as fertilizers. It is the treatment of such effluent with the intent to recover valuable resources (N, P) which is the topic of this paper.

Trickling filters have been used to remove residual COD, nitrogen and phosphorous from high strength anaerobic digester effluent. One study explored nitrification bioreactors to convert nitrogen from swine waste anaerobic digestate (as a proxy for human fecal digestate) (Forbis-Stokes et al., 2018). This study was based on influent COD and total ammonia nitrogen (TAN) concentrations of 1920 mg_COD_ L^−1^ and 500 mg_NH3-H_ L^−1^ and was able to achieve removal efficiencies of 79% and 90%, for COD and TAN respectively (Forbis-Stokes et al., 2018) but inlet concentrations were much lower than those observed in field operation of on-site sanitation systems. Forbis-Stokes et al. also explored denitrification as a potential option to convert NO_3_^−^ obtained from the nitrified effluent to N_2_ gas with NO_3_^−^ and total nitrogen (TN) removal efficiencies of 66 and 45%, respectively (Forbis-Stokes et al., 2018). Uemara et al. evaluated downflow hanging sponge (DHS) performance as a pilot scale post-treatment for upflow anaerobic sludge blanket (UASB) effluent (Uemura et al., 2006). For raw sewage influent concentrations of 33 mg_N_ L^−1^, they observed 70% TAN removal through DHS nitrification and denitrification. The three-year-long experiment demonstrated a stable municipal sewage treatment solution under variable condition (Uemura et al., 2006). Trickling filters were explored for post-treatment of UASB effluent (Chernicharo and Nascimento, 2001). The operating conditions were superficial hydraulic and volumetric organic loading rates of 3.4–30.6 m^3^ m^−2^ day^−1^ and 0.3–3.9 kg_BOD_ m^−3^ day^-1^, respectively. Average COD removal was between 74 and 78%, resulting in final effluent concentration of 60–120 mg_COD_ L^−1^. These studies demonstrate effective removal of TAN and simultaneous removal of organic contaminants from high strength waste streams.

Phosphorus is a pollutant in many waste streams as it can lead to eutrophication of aquatic environments. Phosphorus is also a limited resource and there is growing motivation to explore sustainable ways to source it as it is a critical nutrient for agricultural plant growth (Scholz et al., 2013; Mullins, 2005). Phosphorus can be found in high amounts of human waste up to 1.6–1.7 g person^−1^ day^−1^ and has the potential to precipitate out of solution as struvite (MgNH_4_PO_4_·6H_2_O) which can lead to operational issues in conventional wastewater treatment plants (Schouw et al., 2002). The most common chemical method for wastewater phosphate removal is chemical precipitation with aluminum or iron salts, though such precipitates do not have the same capacity to be used as a fertilizer compared to struvite (Stazi and Tomei, 2018). Struvite precipitation is also best suited for wastes with high phosphorus content such as swine manure, landfill leachate, etc. Since, human waste does not contain high enough concentrations of Mg to precipitate struvite, Mg is often added, generally as MgO, Mg(OH)_2_, or MgCl_2_ to induce struvite precipitation (Barbosa et al., 2016). Although effective, it is often difficult to source such industrial grade chemicals in low resource areas which lack access to safely managed sanitation.

Hybrid anion exchange resins have been used in column tests to precipitate struvite from anaerobic digester filtrate (77 mg P mL^−1^) and urine (619 mg P mL^−1^) up with efficiencies (based on PO_4_-P) of 96.7–99.8% (O'Neal and Boyer, 2013). For anaerobic digestion anaerobic digester effluent with ortho-P concentrations of 61 mg L^−1^, some researchers have added magnesium hydroxide to precipitate struvite with 94% ortho-P removal efficiency.

Agricultural residues and low cost materials could be used as media in biological filtration processes as some have demonstrated practical use for ion exchange and adsorption (Bhatnagar et al., 2015). Of those materials, several seem good candidates for use in effluent treatment and resources recovery from high strength digestate at OSS systems. Biochar is a carbon material, produced from the combustion of plant or organic matter often used as a sorbent in environmental applications because of its high surface area characteristics and economical production (Ahmad et al., 2014; Mohan et al., 2014). Coconut husks could serve as a suitable external electron donor for denitrifiers. Zeolite is a microporous aluminosilicate mineral that is commonly used as a sorbent for environmental applications (Kocatürk-schumacher et al., 2016). Due to the overall negative net charge of their framework, zeolites have a high affinity to adsorb cations, and are particularly selective for ammonium (Beler-Baykal et al., 2011). Beler-Baykal et al. observed ammonium removal rates up to 9.5 mg_NH4_ g^−1^ clinoptilolite, resulting in over 90% removal of ammonium from conventional domestic wastewater (Beler-Baykal et al., 2011). Sunflower is a crop that can be grown in many of the regions around the world where there is also great need for improved sanitation access. Sunflowers seeds have high levels of magnesium content, about 390 mg/100 g (Food Standards Agency Institute of Food Research, 2002) and thus have the potential to serve as a natural source of magnesium for struvite precipitation.

The objective of this study was to quantify the organic and nutrient removal potential of seven materials that can be sourced in poor sanitation access areas and evaluate their suitability as filter media material for biofiltration post-treatment of anaerobic digestion effluent. The vision is that where a liquid fertilizer is valuable, nitrification alone and P recovery as struvite would be used, whereas where N is undesirable, denitrification would be added and water could be reused. This study builds upon previous work (Forbis-Stokes et al., 2018) which was focused on much lower N concentrations and did not consider P recovery. Here, the focus was on the performance of nitrification and denitrification as post-treatment processes for minimally diluted human waste anaerobic digestate and on the treatment of organics, and recovery of nitrogen and phosphorous.

## 2 Materials and methods

### 2.1 Influent waste: excreta digestate production

A lab-scale anaerobic digester was built to simulate field digesters utilized in on-site sanitation treatment systems. The digester had an 80 L capacity and was fed a mixture of urine, feces and water (6.8:2.7:12.8 vol.) at a rate of 22.4 L week^−1^ which resulted in a hydraulic retention time (HRT) of 25 days. The urine, feces, and water were blended together with an industrial submersible blender and poured into the digester inlet. The digester was an unmixed reactor with loading rates of 1.73 kg_COD_ m^−3^ day^−1^, and 0.80 kg_N_ m^−3^ day^−1^. It was operated at room temperature (20 ± 2 °C).

In an effort to best simulate an actual field sanitation system, real feces and urine were used to create the influent stream. Dog feces were used instead of human feces, due to ease of collection and availability. The feces were collected periodically from a local dog boarding center and stored at 4 °C until use. Fresh human urine was collected in a portable urinal with integrated storage placed in our laboratory's men's restroom. Both dog feces and urine were stored for no longer than 4 weeks prior to be fed to the anaerobic digester. Tap water was used to simulate minimal flush water. The digester effluent was deemed representative for AD-based on-site sanitation systems in less developed countries.

After initial start-up and operation of the anaerobic digester, the digester effluent was in average 16,100 mg_COD_ L^−1^ and 2300 mg_N_ L^−1^. Thus, to operate the filter at loading rates similar to field designs, the anaerobic digestion effluent was further adjusted by slight dilution with tap water and more human urine to achieve an average of about 4500 g_COD_ L^−1^ and 3000 g_N_ L^−1^ influent for each of the trickling filters. Adjusting the digestor effluent resulted in concentrations around 4560 mg_COD_ L^−1^ and 2987 mg_N_ L^−1^, with most (96%) of the nitrogen being in the ammonium form.

### 2.2 Filter media

Each filter was filled with 4.86 L of medium. The four trickling filter media types were biochar (BIO), GAC (GAC), coconut husks (COC), and zeolite (ZEO). The four downflow submerged denitrification filter media types were bamboo (BAM), coconut husks (COC), rice husks (RIC), and sunflower seeds (SUN). A wire mesh was placed horizontally between the 10.2 cm ID PVC pipe and the PVC end cap to hold the media up. Media was poured into each filter and compacted every 10 cm.

The biochar for Phase 1 and Phase 2 was made from pine and sourced from Biomass Controls, LLC (Putnam, CT). The biochar for Phase 3 was Charcoal Green Cocochar biochar made from coconut shell and sourced from BuyActivatedCharcoal.com. The GAC for Phase 1–3 was derived from coconut shells and also sourced from BuyActivatedCharcoal.com. Coconut husks were sourced from US Orchid Supplies from Oxnard, California. David brand original roasted and salted sunflower seeds were bought at a local grocery store and used for both trickling and submerged filters. Clinoptilolite zeolite was sourced from Pentair Aquatic Eco-Systems. Bamboo (*Phyllostachys edulis*) was sourced in North Carolina and was shredded to create bamboo chips. The rice husks were sourced from Peaceful Valley Farm & Garden Supply in Grass Valley, California.

### 2.3 Inoculation

Before inoculation, tap water was pumped through the DN filters for 3 days at 10 L day^−1^ to wash out suspended particles and highly soluble salts. Each trickling filter and submerged filter was inoculated once with 1 L activated sludge and 1 L anaerobic digester effluent from a local wastewater treatment plant, fed over the course of 6 h. The inoculum was fed into the inlet port and the mid-point of each trickling and submerged filter.

### 2.4 Experimental setup

**Fig. 1:**
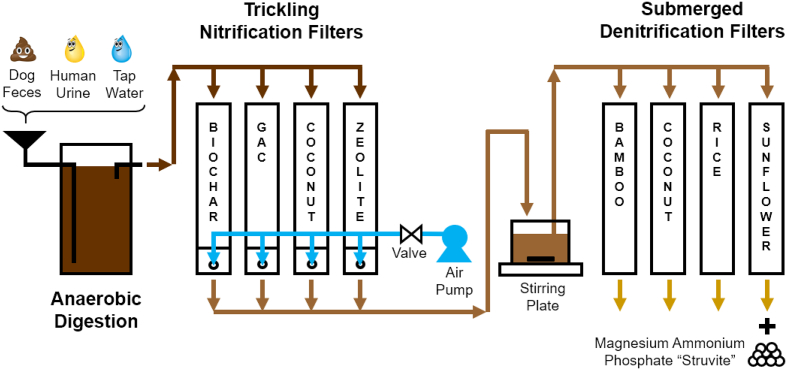
Schematic of nitrification-denitrification post-treatment system.

The filters were initially designed for inlet concentrations of COD and N of 4500 mg_COD_ L^−1^ and 3000 mg_TAN_ L^−1^, respectively. Experiments were conducted in a system of four different trickling filters followed each in series by submerged filters (Fig. 1). Each filter was constructed using 60 cm of 10.2 cm-inner diameter clear PVC pipe and PVC caps (Fig. 1). Holes were drilled in the bottom of each of the eight filters to serve as an end-filter sampling port, and effluent discharge. Each of the four submerged filters had mid-filter sampling ports. Media was supported in each trickling filter with a fine mesh, allowing the liquid waste stream to trickle through the bed and then be collected at the bottom of the filter and pumped into the denitrification filters. Samples were taken from the bottom where the waste stream collected. Below the trickling filter media mesh was a hole in the side of the filter approximately 1.2 cm in diameter, used for natural aeration using the stack effect. After observing oxygen limitation on day 134, air was pumped into the trickling filters at a rate of 10 L min^−1^. The pressure drop was not measured. The tops of the filters were left open and exposed to the laboratory atmosphere. The exterior of each filter was covered in aluminum foil to prevent the growth of photosynthetic bacteria and algae.

The effluents from all nitrification filters were drained into a continuously stirred vessel, which was used to pool the liquid before it was used as the influent to the denitrification filters. The four submerged denitrifying filter media types were bamboo (BAM), coconut husks (COC), rice husks (RIC), and sunflower seeds (SUN). The downflow denitrification filters were similarly constructed except that the media was submerged, and there was no aeration. To keep the beds submerged, each filter's effluent line was routed upwards to an overflow fitted with an air gap, thus ensuring a constant liquid level in the submerged filters. The tops of each filter bed were also covered with a mesh to constrain floating media.

The anaerobic digester effluent (adjusted by slight dilution) was pumped with peristaltic pumps into the top of each trickling filter at a rate corresponding to a volume of 0.8 L day^−1^. The daily feed pattern consisted of multiple 5-minute intervals with a pump timer at various times throughout the day (06:00, 07:00, 08:00, 11:00, 12:00, 13:00, 18:00, 19:00, 20:00, 21:00) to simulate peak flow times. All systems were kept at room temperature (20 ± 2 °C).

### 2.5 Phases

The experimental set up included four different trickling filters each with different nitrification filter media: BIO, GAC, COC, and ZEO, and four different denitrification filter media: BAM, COC, RIC, and SUN. During Phase I, aeration of the nitrification filters was by natural ventilation from the stack effect due to the hole at the bottom of the filter base, as indicated in Table 1. This Phase 1 lasted for 18 weeks for all filters, except the COC medium filter which lasted for the latter 10 weeks. Phase 2 involved the same four trickling filters with BIO, GAC, COC, and ZEO filter media. For this phase, aeration for the nitrification filters was with forced draft air injected at the bottom of each filter at a rate of 10 L min^−1^. This was determined based on the average oxygen demand of the influent COD and TN concentrations (4560 mg L^−1^ and 2987 mg L^−1^, respectively and flow of 0.8 L day^−1^ filter^−1^), this aeration would theoretically be exactly stoichiometric to completely oxidize the contaminants. No changes were made to the experimental conditions of the denitrification filters during Phase 2. The results reflect denitrification filter performance as a result of changes in experimental conditions of the preceeding nitrification filters. After 17 total weeks, the BIO and GAC nitrification filters began to clog and were exchanged for identical but larger-sized fresh media. These experimental changes for Phase 3 were only applicable to the recently changed BIO and GAC media filters. Likewise, with Phase 2, the Phase results of the denitrification filters reflect their performance as a result of changes in experimental conditions of the preceeding nitrification filters (Table 2).

**Table 1:** Sizes of filter media (mm).

	Media	Phase 1	Phase 2	Phase 3
Nitrification filters	BIO	1.5	1.5	4.0
	GAC	0.3	0.3	2.4
	COC	4.8	4.8	4.8
	ZEO	2.4	2.4	2.4
Denitrification filters	BAM	2.0	2.0	2.0
	COC	4.8	4.8	4.8
	RIC	1.2	1.2	1.2
	SUN	4.8	4.8	4.8

**Table 2:** Description of experimental phases.

	Media	Phase 1		Phase 2		Phase 3	
		Aeration	Duration (days)	Aeration	Duration (days)	Aeration	Duration (days)
Nitrification filters	BIO	Natural convection	126	Forced draft	42	Forced draft	23
	GAC	Natural convection	126	Forced draft	42	Forced draft	23
	COC	Natural convection	70*	Forced draft	73	Forced draft	N/A
	ZEO	Natural convection	126	Forced draft	73	Forced draft	N/A
Denitrification filters	BAM	None	126	None	42	None	23
	COC	None	126	None	42	None	23
	RIC	None	126	None	42	None	23
	SUN	None	126	None	42	None	23

* COC medium filter was started after the other filters.

### 2.6 Parameter testing

Starting on Day 0, samples were taken from the outlet of each trickling filters and from the outlet and mid-depth locations of the submerged filters. Total nitrogen (TN), total ammonia nitrogen (TAN or NH_3_-N), nitrite (NO_2_-N), nitrate (NO_3_-N), COD and reactive phosphate (PO_4_-P) were measured weekly using Hach kits (Loveland, Colorado). Dissolved oxygen (DO) and pH were measured using dedicated probes (Hach HQD portable meter with Intellical LDO101 DO sensor, Loveland, CO, and Oakton Instruments Ion 510 series pH meter, Vernon Hills, IL, respectively). Turbidity was measured using EPA Method 180.1 (2100Q Portable Turbidimeter, Loveland, CO), all of which were performed weekly.

### 2.7 X-ray diffraction (XRD)

Identification of struvite was done by XRD on a Panalytical X'Pert Pro. The X-ray generator was set to 45 kV and 40 mA and the Cu K-alpha wavelength = 1.5406 Å. The operating conditions of the 2-theta measurement range were 10–80° and the step size was 0.05°. XRD was performed at the Duke University Shared Materials Instrumentation Facility (SMIF).

## 3 Results

### 3.1 Trickling filters

**Fig. 2:**
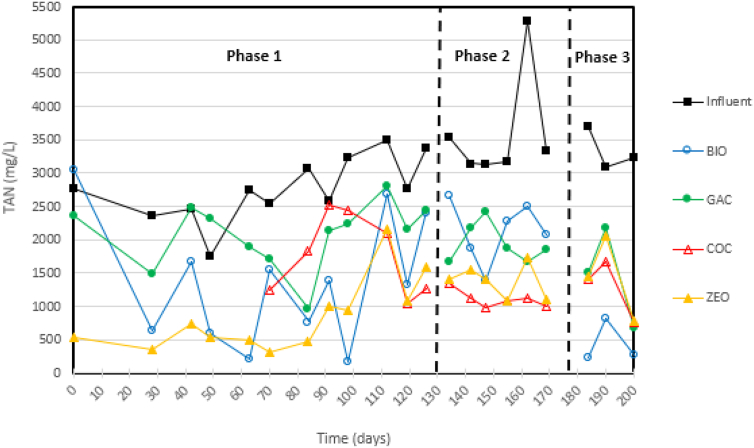
Influent and effluent TAN concentrations of nitrification trickling filters.

**Fig. 3:**
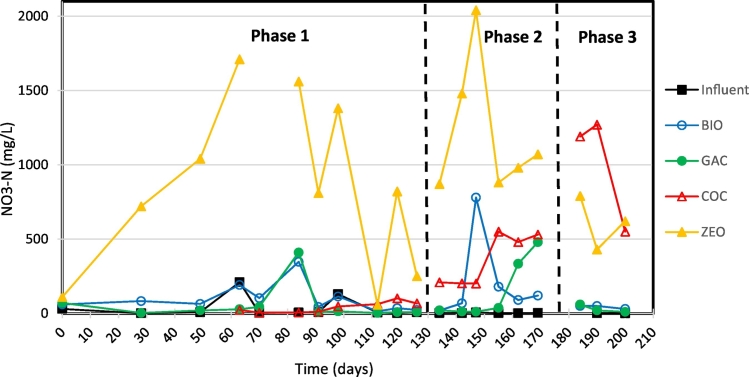
Effluent NO_3_-N concentrations of nitrification trickling filters.

On average, over 96% of the TN in the influent was in the form of NH_4_^+^ thus fit to be converted by nitrification to NO_2_^−^ and NO_3_^−^ in the trickling filters so that, if desired, these species could subsequently be converted to N_2_ gas in the submerged anaerobic filters. During Phase 1, the ZEO medium filter had the highest removal of TAN at 69% compared to 25–50% in the other filters (Fig. 2, Fig. 3). The ZEO filter also yielded the highest TN removal at 39%. During Phase 1, 34% of TN and 36% of NH_3_ were removed in the COC medium filter but only yielded 0.37 mg_NO3-N_ L^−1^ day^−1^. Compared to the 96 mg_NO3-N_ L^−1^ day^−1^ nitrate production in the ZEO filter, the COC medium filter proved to be significantly less efficient. As determined by performing a nitrogen balance with nitrite and nitrate production, these findings suggest that the primary removal mechanism for TN and NH_3_ removal for the trickling filter systems was adsorption. Long term, as filter media adsorption sites become saturated, filters will have to be exchanged for fresh media.

During Phase 2, air was pumped into the bottom of each filter to increase oxygenation necessary for nitrification. The nitrite and nitrate production of all filters increased, suggesting that the oxygen was limiting nitrification under natural draft aeration operating conditions. Aeration had the greatest effect on the COC medium filter as the NO_3_-N production rate increased from 0.0004 kg m^−3^ day^−1^ in Phase 1 to 0.075 kg m^−3^ day^−1^ in Phase 2. This suggests that nitrification became the more prominent NH_3_ removal mechanism as aeration increased. Similarly, the ZEO medium filter's NO_3_-N production rate increased from 0.096 to 0.134 kg_NO3-N_ m^−3^ day^−1^. In contrast, introducing pumped aeration decreased TAN removal in both BIO and ZEO media filters by 9% each. As increased aeration resulted in an increase in COD removal, it is suspected that these maintained a higher concentration of heterotrophic bacteria and the increase in aeration further increased competition for the nitrifiers.

In Phase 1, the BIO medium filter removed more TN than the GAC medium filter, but in Phase 2 the GAC filter removed more TN than the BIO filter (Table 3, Table 4). During Phase 2, the GAC filter had a higher ammonium-nitrate conversion rate than the BIO filter. This change can likely be attributed to the amount in DO in each filter. In Phase 1, the BIO medium filter had an average of 2.58 mg_DO_ L^−1^ while the GAC medium filter had an average of 1.7 mg_DO_ L^−1^. In Phase 2, the BIO and GAC filters had 1.03 mg_DO_ L^−1^ and 3.80 mg_DO_ L^−1^, respectively. The change can also be explained by the changes in pH, as an indicator for nitrification. From Phase 1 to Phase 2, the BIO medium filter pH increased from 7.90 to a less optimal 8.35. The increase in DO in the BIO medium filter also aided heterotrophic growth which may have outgrown the nitrifiers, as COD removal increased by 16% while NH_3_ removal decreased by 9%.

**Table 3:** Nitrogen speciation in the trickling filters influent (INF) and effluent. The Δ indicates removal or change with respect to the influent (INF).

		TN (mg L^−1^)			NH_3_-N (mg L^−1^)			NO_3_-N (mg L^−1^)		NO_2_-N (mg L^−1^)	
		Avg	St. dev	Δ (%)	Avg	St. dev	Δ (%)	Avg.	St. dev	Avg.	St. dev
Phase 1	INF	2852	701	–	2764	463	–	37	65	1	1
	BIO	2157	858	24%	1370	916	50%	98	92	274	209
	GAC	2280	420	20%	2082	480	25%	56	114	91	118
	COC	1875	724	34%	1780	559	36%	40	33	187	215
	ZEO	1751	836	39%	850	525	69%	846	558	104	81
Phase 2	INF^a^	3267	309	–	3598	766	–	3	2	2	2
	BIO	2667	236	18%	2128	425	41%	210	260	510	189
	GAC	2033	137	38%	1943	276	46%	149	187	198	122
	INF^b^	3167	374	–	3512	655	–	2	2	1	2
	COC	2200	537	24%	1170	254	67%	576	378	361	151
	ZEO	2389	708	30%	1398	353	60%	1018	455	271	138
Phase 3	INF	2967	411	–	3340	263	–	1	0	0	0
	BIO	933	450	69%	437	264	87%	43	9	4	4
	GAC	1733	403	42%	1450	614	57%	30	22	0	0

a Phase 2 influent for BIO and GAC filters.

b Phase 2 influent for COC and ZEO filters.

**Table 4:** Characteristics of trickling filters influent and effluent. The Δ indicates removal or change with respect to the influent (INF).

		COD (mg L^−1^)			pH		Turbidity (mg L^−1^)			PO4-P (mg L^−1^)		
		Avg	St. dev	Δ (%)	Avg	St. dev	Avg.	St. dev	Δ (%)	Avg.	St. dev	Δ (%)
Phase 1	INF	4611	1351	–	9.20	0.43	5890	5002	–	1368	1245	–
	BIO	2049	3241	56%	7.90	0.61	40	38	99%	435	79	68%
	GAC	1529	1196	67%	8.75	0.28	170	142	97%	451	75	67%
	COC	1719	771	63%	7.90	0.96	261	411	96%	546	126	60%
	ZEO	1924	2304	58%	7.40	0.61	317	291	95%	232	143	83%
Phase 2	INF^a^	4727	2197	–	9.40	0.16	1978	1527	–	1160	805	–
	BIO	1318	204	72%	8.35	0.55	76	48	96%	505	87	56%
	GAC	742	349	84%	8.71	0.43	72	37	96%	447	87	61%
	INF^b^	4589	1924	–	9.30	0.39	1904	1740	–	1130	762	–
	COC	1783	593	61%	7.06	0.80	67	85	96%	787	172	30%
	ZEO	1319	567	71%	7.42	0.76	163	162	91%	294	117	74%
Phase 3	INF	4313	1158	–	9.18	0.54	1807	1986	–	1070	666	–
	BIO	9827	8603	−128%	8.86	0.19	3009	2881	−67%	554	272	48%
	GAC	1490	875	65%	9.39	0.03	483	201	73%	637	137	40%

a Phase 2 influent for BIO and GAC filters.

b Phase 2 influent for COC and ZEO filters.

From Phase 1 to Phase 2, PO_4_-P removal in the COC medium filter significantly decreased from 60% removal to 30% removal. This could be a result of the primary mechanism of phosphorus removal being adsorption and the change could be a result of saturation rather than biological removal, as bio-P removal is typically incorporated into cell biomass and removed by sedimentation. The efficiency of the ZEO medium filter decreased from 83 to 74%. This could be indicative of the beginning of saturation for filtration or adsorption. There were no significant changes in PO_4_-P removal for the BIO and GAC media filters.

### 3.2 Denitrifying filters

Overall, the COC submerged filter (Phase 1) performed the best with 21% COD removal, 30% TAN removal, and 71% PO_4_-P removal. During Phase 1, the BAM, COC, RIC, and SUN media filters yielded COD removal efficiencies of −13, 21, −21, and −364%, respectively. For the BAM, RIC and SUN submerged denitrification filters, the COD concentration was higher in the effluent than the influent because of organic matter leaching into the waste stream undergoing treatment. Thus, electron donors were not the limiting factor for denitrification in these RIC and SUN submerged filters.

Each filter exhibitted increased COD efficiencies in Phase 2 with 32, 26, 23, and −75%, respectively. This indicates that less organics were being leached or more organics were being biologically consumed. Also, for Phase 2, the average COD concentration entering the denitrifying filters was 1277 mg L^−1^ which is less than half of the COD concentration entering during Phase 1 at 2818 mg L^−1^, as seen in Table 6. This is consistent with the hypothesis that oxygen was rate limiting in the aerobic filters with natural convection aeration in Phase 1. Conversely, the DO levels could have slightly inhibited the performance of the subsequent denitrification filters. For the BAM, COC, and RIC media filters, DO averaged 0.69–0.76 mg_DO_ L^−1^, 0.83–0.89 mg_DO_ L^−1^, 0.84–0.92 mg_DO_ L^−1^, respectively for all three operational Phases. The SUN medium filter yield more favorable anoxic conditions and averaged 0.10–0.12 mg_DO_ L^−1^, which could also explain higher denitrification rates. Furthermore, in Phase 2, the submerged denitrification filters removed significantly higher COD than in Phase 1. This result could also be indicative of the reduced COD loading rate being more favorable to the denitrification process under the designed experimental conditions.

BAM and RIC media filters achieved 66% and 65% removal of PO_4_-P, respectively, while the SUN medium filter removed 38%. Shifting from Phase 1 to Phase 2, COD removal efficiency increased in all filters and simultaneously became less efficient at removing PO_4_-P, with exception of the SUN medium filter. This was most apparent in the COC medium filter as performance decreased to 51% removal efficiency. This could further support that the primary removal mechanism of PO_4_-P is adsorption. The RIC filter during Phase 1 had a COD removal efficiency of −21% and a TN removal efficiency of 30%. For Phase 2 and Phase 3 it had COD removal efficiencies of 23% and 40%, respectively, and TN removal efficiencies of 9% and 13%, respectively.

**Fig. 4:**
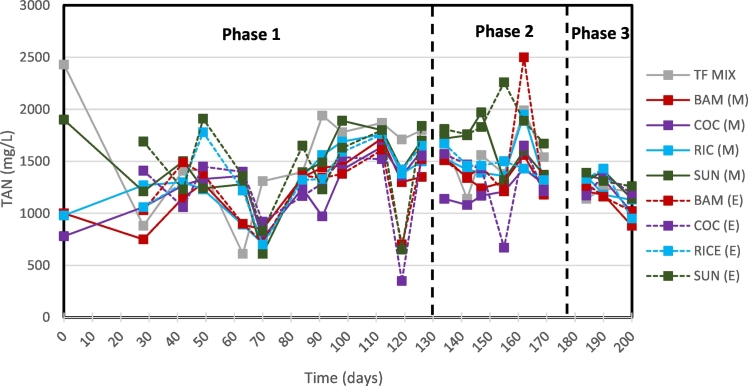
Mid-point (M) and end-point (E) total ammonia concentrations of denitrification submerged filters.

**Fig. 5:**
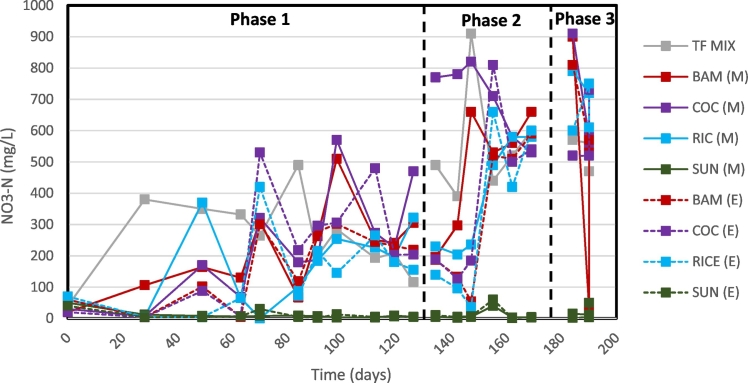
Mid-point (M) and end-point (E) nitrate concentrations of denitrification submerged filters.

The BAM, COC, and RIC filters for Phase 1 had the highest TN removal efficiency of 30% (Fig. 4, Fig. 5, Table 5). A portion of this TN removal is attributed to NO_3_-N removal (28–44%) and NO_2_-N removal (50–56%) by denitrification. Interesting though, these three filters also yielded NH_3_ removal at 11–21%. This suggests that the submerged denitrification filters are continuing to adsorb NH_3_. However, throughout each Phase, the RIC filter had similar NO_2_-N removal efficiencies (54–61%). From Phase 1 to Phase 2, the RIC medium filter also yielded similar NO_3_-N removal efficiencies at 44% and 42%, respectively, yet increased COD removal efficiencies from −21% to 23%, respectively. In Phase 3, RIC filter's NO_3_-N efficiency dropped to −23%. The SUN filter, for each Phase, was able to remove almost all the NO_2_-N and NO_3_-N in the influent between 96 and 99%.

**Table 5:** Nitrogen speciation of the submerged denitrification filter influent (INF) and effluent. The Δ indicates removal or change with respect to the influent (INF).

		TN (mg L^−1^)			NH_3_-N (mg L^−1^)			NO_3_-N (mg L^−1^)			NO_2_-N (mg L^−1^)		
		Avg.	St. dev	Δ (%)	Avg.	St. dev	Δ (%)	Avg.	St. dev	Δ (%)	Avg.	St. dev	Δ (%)
Phase 1	INF	2113	619	–	1546	467	–	260	121	–	226	148	–
	BAM	1470	476	30%	1221	310	21%	170	109	35%	113	103	50%
	COC	1470	467	30%	1241	344	20%	214	172	18%	100	104	56%
	RIC	1487	560	30%	1375	306	11%	145	118	44%	103	120	54%
	SUN	1597	466	24%	1438	398	7%	11	12	96%	1	3	99%
Phase 2	INF	2150	443	–	1552	262	–	558	170	–	406	78	–
	BAM	1650	214	23%	1533	448	1%	332	213	40%	117	96	71%
	COC	1783	261	17%	1333	326	14%	392	246	30%	167	88	59%
	RIC	1950	222	9%	1473	104	5%	325	247	42%	182	87	55%
	SUN	1800	327	16%	1870	187	−21%	15	21	97%	0	0	100%
Phase 3	INF	2000	245	–	1197	42	–	533	45	–	233	129	–
	BAM	2167	478	−8%	1157	110	3%	637	124	−19%	60	44	74%
	COC	1767	94	12%	1243	90	−4%	590	99	−11%	79	56	66%
	RIC	1733	665	13%	1237	207	−3%	653	68	−23%	92	67	61%
	SUN	1533	1066	23%	1320	54	−10%	5	3	99%	0	0	100%

**Table 6:** Characteristics of the submerged denitrification filter influent (INF) and effluent. The Δ indicates removal or change with respect to the influent (INF).

		COD (mg L^−1^)			pH		Turbidity (mg L^−1^)			PO4-P (mg L^−1^)		
		Avg	St. dev	Δ (%)	Avg	St. dev	Avg.	St. dev	Δ (%)	Avg.	St. dev	Δ (%)
Phase 1	INF	2818	3501	–	8.34	0.55	536	253	–	532	378	–
	BAM	3189	5335	−13%	8.47	0.24	77	51	86%	180	50	66%
	COC	2239	3034	21%	8.45	0.21	35	14	93%	154	55	71%
	RIC	3400	5844	−21%	8.50	0.19	72	42	87%	184	57	65%
	SUN	13,080	24,186	−364%	7.09	0.39	210	133	61%	331	78	38%
Phase 2	INF	1277	204	–	8.14	0.38	461	314	–	565	206	–
	BAM	872	160	32%	8.47	0.29	16	2	97%	292	34	48%
	COC	943	159	26%	8.42	0.46	17	2	96%	279	30	51%
	RIC	980	202	23%	8.62	0.23	17	1	96%	290	49	49%
	SUN	2239	1594	−75%	7.08	0.59	59	10	87%	310	123	45%
Phase 3	INF	1820	368	–	8.69	0.21	402	314	–	627	27	–
	BAM	1143	111	37%	8.44	0.05	19	1	97%	406	55	35%
	COC	1010	106	45%	8.45	0.09	15	3	96%	362	70	42%
	RIC	1093	95	40%	8.55	0.02	16	3	96%	376	58	40%
	SUN	4837	276	−166%	7.28	0.34	80	10	87%	383	32	39%

### 3.3 Phosphorus precipitation & recovery

After about 35 days of operation, it was observed that a white crystal precipitate was forming in the denitrification SUN medium filter effluent tubing. Samples were extracted from the submerged SUN filter outlet port and dried in a furnace at 550 °C for 1 h. X-ray diffraction analysis was performed to characterize the crystalline solid. Analysis yielded presence of ammonium magnesium phosphate hydrate and ammonium magnesium hydrogen phosphate hydrate. Ammonium magnesium phosphate, also known as struvite, has been demonstrated to be a slow-release nutrient fertilizer that is useful for agricultural purposes.

## 4 Discussion

### 4.1 Operational challenges

The first phase of the experiments relied on aeration by natural convection. However, after about 12 weeks, oxygen became limiting and the DO concentration of the filter effluent started to decrease from 3.6–4.8 mg L^−1^ to 0.1–1.9 mg L^−1^. In addition to the simultaneous clogging of the BIO and GAC media filters, it was determined that the natural air flow into the filter was not enough for all the fast-growing heterotrophic bacteria and autotrophic nitrifying bacteria. It was then decided to pump air into the filters (i.e. Phase 2).

Initially, in Phase 1, the BIO and GAC filter media were used. At the beginning of Phase 1, the influent was able to trickle all the way through the filter. However, after about 14 weeks, the filters started to clog at the top. Because of the small effective media diameter of 1.5 mm and 0.3 mm, the biochar and GAC also served as physical filter for suspended solids in the influent. To declog the filters, a layer of media and influent solids, about 1 cm thick, was removed from the top of the filter, and operation was resumed. Clogs then soon started to be a reoccurring issue, about every week. Thus, it was decided to replace the BIO and GAC filter media with larger effective sized diameter media of 4.0 mm and 2.4 mm, respectively, such that the filters would serve less as a physical filter and more as a biological filter.

### 4.2 Choice of material

It was initially thought that using sunflower seeds as a trickling filter media would be good to produce struvite because of their high magnesium content. Sunflower seeds are a cheap, sustainable crop that can be grown in many places around the world. Interestingly, sunflower seeds have some of the highest magnesium concentrations of all foods. When a 1:1:1 ratio of NH_4_^+^, PO_4_^3−^ and Mg^2+^ are combined, struvite is formed. Since most of the nitrogen species in the trickling filter is in the form of NH_4_^+^, it was thought that the trickling filter would be a good place for struvite formation.

After the start of the experiment, a lot of the organic matter from the sunflower seeds (seed and shell) was leaching into the treated wastewater, particularly in the later phases. This resulted in higher average COD effluent concentration than influent concentration, 2818 mg_COD_ L^−1^ and 13,080 mg_COD_ L^−1^, respectively in Phase 3. After about 4 weeks of operation, flies started to appear in the laboratory and the sunflower seed trickling filter sample port became clogged with maggots. Flies were not present in other filters. The sunflower seed trickling filter was decommissioned and replaced with coconut husks (same as in denitrification filters). Although, the temporary sunflower seed filters promoted some nitrification, using sunflower seeds as trickling filter medium would not be practical for field use because of the excessive leaching of organics. This experience suggests that other media which contain readily biodegradable materials should not be used.

Similarly, the readily biodegradable materials were thought to be a cause of concern for the respective submerged denitrification filter. The sunflower seed and rice husk submerged denitrification filter yielded negative COD removal, as the rate of organics leaching into the wastewater was greater than the biological organic consumption rate, though the sunflower seed submerged filter was the only one to precipitate ammonium magnesium phosphate. It may be more practically feasible to combine sunflower seeds with other media type to achieve both denitrification and organic matter removal. However, in the context that high organic matter in effluent is less of an issue, sunflower seed medium submerged denitrification filters could serve as a very effective option to remove almost all NO_2_-N and NO_3_-N loading. With removal rates of 0.037–0.068 kg_NO2_ m^−3^ day^−1^ and 0.041–0.091 kg_NO3_ m^−3^ day^−1^, the SUN medium denitrification filters removed 99% and 96–99% of influent NO_2_-N and NO_3_-N, respectively.

### 4.3 Practical applications

Experimental data from laboratory operation have demonstrated combined removal of COD, TN, and PO_4_-P up to 84%, 69%, and 89%, respectively, partly meeting the treatment requirements (80% load reduction for P and 70% for N) of a newly published ISO standard (30500) for non-sewered sanitation systems. However, the effluent is not polished enough to meet discharge or reuse standards completely, thus optimization of post-treatment processes is necessary to meet standards.

In a practical application where nutrient removal is prioritized, the ZEO medium filter with natural aeration (Phase 1) yielded the highest overall nutrient removal performance with TAN and PO_4_-P removal rates of 0.252 kg_TAN_ m^−3^ day^−1^ and 0.15 kg_P_ m^−3^ day^−1^, respectively. This is high compared to the 0.102 kg_TAN_ m^−3^ day^−1^ removal rate achieved by Forbis-Stokes et al. (2018) for zeolite nitrification trickling filter treatment of swine waste anaerobic digestate. The higher TAN removal rates could be attributed to the higher TAN loading rates from this study (0.364–0.473 kg_TAN_ m^−3^ day^−1^) compared to the Forbis-Stokes et al. (2018) study (0.085–0.123 kg_TAN_ m^−3^ day^−1^). The 196% increase in TAN loading rate (because of higher TAN concentrations) yielded a 147% increase in removal rate indicating positive dependency of nitrification rates with the TAN concentration at the conditions of the experiment. From the COC medium denitrification filter during Phase 1, the nitrification-denitrification filter system was able to achieve a PO_4_-P removal efficiency of 89%. The ZEO medium filter also yielded 58% COD removal at 0.35 kg_COD_ m^−3^ day^−1^. Based on the observed removal rates, an actual sanitation system for a family of 5 would need the greater of 160, 248, and 97-liter filter packed with ZEO to remove close to 100% of COD, TN, and PO_4_-P, respectively from their anaerobically digested human wastes. Thus sizing (248 liter) would be determined by TN removal.

In a context where organic contaminant removal is prioritized, it is recommended that the GAC filter with forced aeration (Phase 2) be used as it yielded the highest COD removal at 84%. The cost implications of such aeration, while moderate, would need to be considered. The GAC filter also supported relatively high TAN and PO_4_-P removal rates of 0.22 kg_TAN_ m^−3^ day^−1^ and 0.094 kg_P_ m^−3^ day^−1^, respectively.

Throughout the duration of the study, the COC and ZEO filter media did not have to be changed. However, the BIO and GAC filter media began to experience clogging after around 12 weeks of operation. If BIO or GAC media trickling filters are to be operated in field conditions, it is recommended that larger diameter media be used to avoid quarterly media replacement.

## 5 Conclusion

The fertilizer-generating anaerobic digestion post-treatment filter system simultaneously addresses the global lack of access to improved sanitation and could potentially expand the capacity for small-scale agricultural development when combined with on-site sanitation technologies like the Anaerobic Pasteurization Latrine (ADPL) (Forbis-Stokes et al., 2016). Piloted in Kenya, India, and the Philippines, the ADPL system operates by gravity flow, requires little maintenance, is constructed using local resources, and provides self-contained and energy-neutral on-site sanitation using anaerobic digestion of human wastes to generate biogas as fuel to pasteurize the treated effluent. The nitrification and denitrification post-treatment filters discussed herein, when used in conjunction with the ADPL or other on-site sanitation technologies, have the greatest potential in geographic areas with poor access to sanitation, declining national food security, water scarcity during the dry seasons, and are most vulnerable to climate change and extreme weather events (Hunter et al., 2018; Paun et al., 2018).

## Declaration of competing interest

We wish to confirm that there are no known conflicts of interest associated with this publication and there has been no significant financial support for this work that could have influenced its outcome.

## Appendix A Supplementary data

Supplementary data include X-ray diffraction spectrum and associated table with chemical composition of precipitate. Image 1
