# Supplementary material for: Resources recovery from high-strength human waste anaerobic digestate using simple nitrification and denitrification filters
Source: Sci Total Environ. 2020 Apr 10;712:135509. doi: 10.1016/j.scitotenv.2019.135509 (PMC7014583; doi:10.1016/j.scitotenv.2019.135509)
Supplement: Supplementary file 1 — Supplementary data include X-ray diffraction spectrum and associated table with chemical composition of precipitate. [file mmc1.docx]

**Supplementary Information for:**

**Resources Recovery from High-Strength Human Waste Anaerobic Digestate Using Simple Nitrification and Denitrification Filters**

Brandon Hunter, Marc A. Deshusses

Dept. of Civil and Environmental Engineering

127C Hudson Hall; Box 90287

Duke University

Durham, NC 27708-0287. USA

(919) 660-5480; (919) 660-5219 FAX

[marc.deshusses@duke.edu](mailto:marc.deshusses@duke.edu)

*X-Ray Diffraction*

Figure A.1: X-ray diffraction spectrum

Table A.1: Scores of precipitant XRD chemical composition matches
